# Heterogeneous influence of neighborhood features on community satisfaction: a comparative study in Beijing’s urban and suburban communities

**DOI:** 10.3389/fpubh.2023.1288868

**Published:** 2023-11-09

**Authors:** Huaxiong Jiang, Runting Cai, Ding Yang, Zhuo Huang, Jinping Song

**Affiliations:** Faculty of Geographical Science, Beijing Normal University, Beijing, China

**Keywords:** community satisfaction, urban and suburban communities, neighborly interactions, urban planning and policies, Beijing

## Abstract

**Introduction:**

Community satisfaction contributes to urban planning, community development, and policy formulation. Yet, we lack comprehensive knowledge about how different neighborhood features impact satisfaction, especially across diverse community types.

**Methods:**

Relied on a sample of 4,009 respondents in Beijing, this study examines the influence of neighborhood features on community satisfaction through neighborly interactions, focusing on the heterogeneity between urban and suburban communities, using structural equation models.

**Results:**

The results reveal that community service and community management exert significant influences on community satisfaction, primarily mediated by the role of neighborly interactions. Then, transportation convenience positively influences community satisfaction in urban areas, while no housing property has a negative effect in suburban communities.

**Discussion:**

These results highlight varied neighborhood effects on community satisfaction, informing tailored urban planning and policies that address unique traits and requirements of different communities.

## 1. Introduction

The concept of community satisfaction has gained significant attention in recent years due to its implications for individual well-being and the overall health of societies (1–3). The sustainable development goals (SDGs) of the United Nations encompass economic, social, and environmental aspects, including both challenges to overcome and new paths created for future sustainable development (4, 5). SDG 10 calls for making cities and human settlements inclusive, safe, resilient and sustainable (6). In China, high-quality development, including the improvement of community residents’ satisfaction, has become a new trend in urban development. Neighborhoods, as the fundamental building blocks of communities, provide a physical and social environment that directly influences residents’ satisfaction with their living conditions and the achievement of those sustainable goals (7).

Numerous studies have explored the relationship between neighborhood features and community satisfaction (8–13). Typically, the “environmental-social model” integrates environmental and social factors to examine how they interact and impact each other. It looks at the physical environment, including safety, access to amenities, green spaces, and infrastructure quality, emphasizing how these affect human health and quality of life (14–16). For instance, Ciorici and Patricia found positive associations between neighborhood satisfaction and the quality of the physical environment, access to amenities, and social cohesion in the North Camden (17). Perceived safety is another key determinant, especially for women and the older adult, as shown by Kuo and Sullivan (18–20). Factors like crime rates, physical disorder, and social disorder shape safety perceptions (21, 22). Social relationships and capital are also crucial for neighborhood satisfaction. Sampson et al. discovered positive links between neighborhood social capital, trust, social networks, and satisfaction, as well as outcomes like reduced crime and increased civic engagement (23).

However, despite the existing body of research, our understanding of the influence of neighborhood features on community satisfaction still has critical gaps. Firstly, most studies tend to focus solely on the neighborhood level without considering the broader urban context. It is important to acknowledge that neighborhood satisfaction is influenced by factors beyond the neighborhood boundaries, such as the larger city or region in which the neighborhood is situated (24–27). Taking into account these wider spatial variables and scales can provide a more comprehensive understanding of community satisfaction.

Secondly, there is a lack of research investigating the heterogeneous influence of neighborhood features on community satisfaction, particularly when comparing urban and suburban communities. Urban and suburban areas exhibit distinct characteristics in terms of population density, built environment, and social dynamics, which can result in different experiences and perceptions of neighborhood features among residents (28). Examining these variations is crucial to uncovering the nuanced effects of neighborhood features on community satisfaction. Additionally, marginalized populations, such as low-income or minority residents, often face unique challenges that can significantly impact their satisfaction with their neighborhoods (29–31). It is essential to include these populations in research to ensure a more inclusive and representative understanding of community satisfaction.

In this study, we focus on Beijing as a unique case study due to its diverse urban and suburban landscape. Rapid urbanization and socio-economic transformations in Beijing have resulted in significant variations in neighborhood characteristics between urban and suburban areas (32, 33), because of unique geographical, social, and economic characteristics. Our research aims to identify and analyze the influence of multiple neighborhood features on community satisfaction via neighborly interactions, with a particular emphasis on the heterogeneous role of these features in urban and suburban communities. We utilize structural equation models to analyze the data obtained from a large-scale survey conducted in Beijing, involving 4,009 respondents. The survey was conducted between May 2019 and September 2020, encompassing a substantial timeframe. It seeks to uncover the influence of neighborhood features beyond the immediate vicinity, explore the mediating role of neighborly interactions, and discern the differential impact of these factors in urban and suburban communities. The findings will enhance our understanding of the factors shaping community satisfaction in urban and suburban areas, offering valuable insights for policymakers, urban planners, and community development practitioners aiming to create more livable and satisfying neighborhoods.

## 2. Literature review

### 2.1. Community satisfaction

Community satisfaction holds significant importance for individuals, communities, and society as a whole. Community satisfaction directly impacts residents’ quality of life (7). When individuals are satisfied with their neighborhoods, they tend to experience higher levels of well-being, happiness, and overall life satisfaction (11, 34). Satisfied communities foster a sense of belonging and social cohesion among residents (35). When individuals feel satisfied with their neighborhood, they are more likely to develop social connections, engage in community activities, and establish meaningful relationships with their neighbors (17, 36). In urban and community planning, community satisfaction is closely tied to sustainability (37). SDGs 11, within the framework of the United Nations Sustainable Development Goals, advocates for the creation of inclusive, safe, resilient, and sustainable cities and human settlements, ultimately aiming to attain a satisfactory and conducive environment for all (6). Satisfied communities often prioritize environmentally friendly practices, community resilience, and resource efficiency, promoting residents to embrace sustainable initiatives such as recycling, energy conservation, public transportation, and green infrastructure (17, 38, 39).

### 2.2. Factors influencing community satisfaction

Community satisfaction is influenced by a multitude of factors. These factors can vary depending on the context, cultural norms, and individual preferences (40). Urban sociology offers valuable insights into the social dynamics within neighborhoods and their impact on community satisfaction. Concepts such as social capital (12), social cohesion (35, 41), and sense of community (42) provide theoretical frameworks to understand the factors influencing community satisfaction.

Physical neighborhood features play a significant role in shaping community satisfaction. These features encompass various aspects, including walkability, access to amenities, green spaces, and the overall aesthetic quality of the environment. For instance, neighborhoods with well-connected sidewalks, pedestrian-friendly infrastructure, and proximity to key destinations such as shops, schools, and parks tend to promote a sense of community satisfaction (43). Residents appreciate the ability to engage in active transportation, socialize with neighbors during walks, and have easy access to essential services and recreational opportunities (44, 45). The availability of essential services such as grocery stores, healthcare facilities, educational institutions, recreational facilities, and cultural spaces within a neighborhood positively contributes to residents’ quality of life and overall satisfaction. Easy access to these amenities reduces the need for extensive travel and enhances the convenience and well-being of residents (17, 46). The presence of green spaces and the overall aesthetic quality of the neighborhood environment also play a significant role in community satisfaction. Well-maintained parks, gardens, and open spaces provide opportunities for recreation, relaxation, and social interactions. These natural and aesthetically pleasing elements contribute to residents’ sense of well-being and satisfaction with their neighborhood (38, 47). A visually appealing neighborhood with attractive architecture, clean streets, and a pleasant ambiance enhances residents’ pride in their community and fosters a positive perception of their surroundings (48, 49).

While many studies have focused on individual and neighborhood-level factors that influence community satisfaction, it is important to consider the broader social, economic, and environmental contexts that shape residents’ experiences and perceptions. Community satisfaction is influenced by factors beyond the neighborhood boundaries (28, 50, 51), such as the larger city or region in which the neighborhood is situated. Taking into account these wider spatial scales can provide a more comprehensive understanding of community satisfaction. Economic conditions, such as employment opportunities, income levels, and poverty rates, can impact residents’ overall satisfaction with their neighborhoods. Neighborhoods with higher levels of economic inequality or limited access to resources may struggle to meet residents’ needs and contribute to lower levels of satisfaction (12, 52). Other factors such as access to city-wide amenities, transportation infrastructure, and employment opportunities may play a significant role in shaping residents’ satisfaction with their neighborhoods (17). For instance, amenities like healthcare facilities, fitness centers, and green spaces contribute to the physical and mental well-being of residents. Access to these resources can improve overall health outcomes and lead to higher levels of satisfaction within a community (11, 47, 53). Then, communities with a diverse range of employment sectors are more economically resilient. A broad job market can withstand economic downturns better, reducing the impact of recessions on community well-being (54–56).

Moreover, the policy and governance context within which neighborhoods operate can influence community satisfaction (57). For example, effective and inclusive governance or management structures that prioritize resident input and address community needs are more likely to foster neighborhood satisfaction and promote positive outcomes (58, 59).

The current literature merely suggests that these features can directly influence community satisfaction. However, they can indirectly shape neighborly interactions by providing opportunities for social gatherings and facilitating shared activities, thereby enhancing community satisfaction (60). For instance, social psychology theories shed light on the cognitive and affective processes underlying community satisfaction. The concept of social identity and in-group/out-group dynamics help explain how neighborhood interactions influence residents’ sense of belonging and satisfaction with their community (61). By facilitating social exchanges, connections, and engagement among residents, neighborhood interaction acts as a mechanism through which neighborhood features influence residents’ satisfaction levels (62). Positive neighborhood interactions contribute to the development of social networks, trust, and social capital within the community (60). They promote a sense of belonging and attachment to the neighborhood, creating a supportive and inclusive environment (63). These interactions can enhance residents’ well-being, provide emotional and instrumental support, and create opportunities for social engagement and the exchange of resources (62).

Neighborhood interactions also play a vital role in community building and problem-solving (64, 65).They facilitate the sharing of information, resources, and knowledge among neighbors, enabling collective action and addressing common concerns or challenges (69). Through collaboration and cooperation, neighbors can work together to improve the quality of their neighborhood, address safety issues, enhance community services, and create a more vibrant and cohesive social environment (53). Effective neighborhood interactions could foster a sense of community and promoting positive outcomes such as improved social relationships, and a stronger sense of collective identity (35). However, the mediation effect of neighborhood interactions on community satisfaction is often underestimated in the existing literature. While neighborhood features have been studied in isolation (10, 13), their combined influence on community satisfaction through neighborly interactions is not extensively explored.

Studies have overlooked the nuanced and diverse influence of neighborhood features across different types of communities. Urban and suburban communities exhibit significant variations in population density, built environment, social networks, and access to amenities and services (51). These differences can shape residents’ experiences, preferences, and satisfaction with their neighborhoods (28, 50). In reality, urban communities may prioritize factors such as proximity to public transportation, cultural institutions, and employment opportunities (67), whereas suburban communities may place greater emphasis on larger homes, green spaces, and a quieter atmosphere (68). Failing to recognize these variations can result in a limited understanding of the factors that contribute to community satisfaction in different contexts, leading to difficulties in informing government decision-makers and community planners to improve the community environment and the quality of life of residents.

### 2.3. Defining the study’s purpose

**Figure 1:**
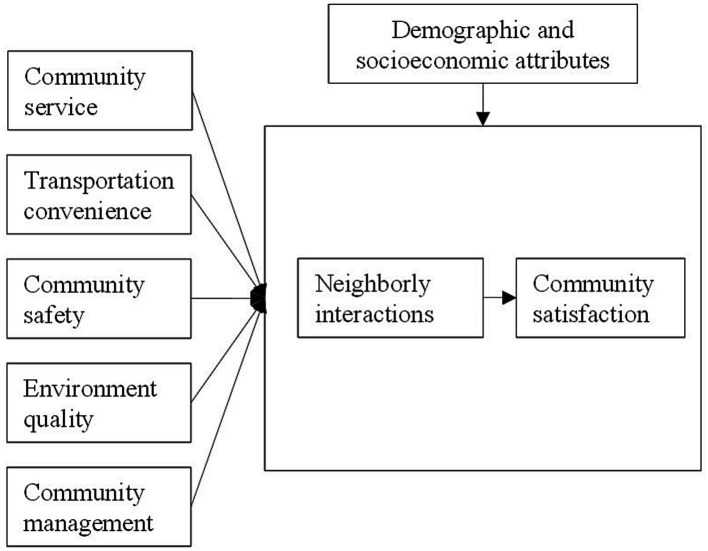
Conceptual framework.

This study seeks to bridge the existing knowledge gap by examining the influence of various neighborhood features on community satisfaction, particularly by considering the role of neighborly interactions. It specifically aims to explore the heterogeneous nature of these features in urban and suburban communities. This study is driven by three primary objectives, as shown in the conceptual framework of Figure 1.

Firstly, it seeks to identify neighborhood features that extend beyond the boundaries of a neighborhood, yet significantly influence community satisfaction. By expanding the scope of analysis beyond the immediate neighborhood context, this research aims to uncover the broader factors such as environment quality and transportation convenience that shape residents’ satisfaction with their community. Secondly, the study aims to examine the mediating role of neighborly interactions in the relationship between neighborhood features and community satisfaction. It recognizes the potential for neighborly interactions to act as a mechanism through which neighborhood characteristics impact residents’ perceptions and overall satisfaction. Lastly, the research aims to compare and analyze the findings between urban and suburban communities. By examining these distinct community types separately, the study seeks to reveal any contrasting patterns or differences in the impact of neighborhood features on community satisfaction. This comparative analysis will shed light on the specific dynamics and factors that contribute to satisfaction within urban and suburban contexts. By addressing these three objectives, the study aims to enhance our understanding of the multifaceted nature of community satisfaction.

## 3. Methodology

### 3.1. Data collection

The choice of Beijing as a case study in this research is likely due to several reasons. Firstly, Beijing is a highly populated and diverse city that represents the characteristics of many urban areas around the world. By selecting Beijing, the study can capture the dynamics of urban and suburban communities in a context that is relevant to a large number of cities globally. Secondly, Beijing offers a unique opportunity to compare and contrast the influence of neighborhood features on community satisfaction between urban and suburban areas. The city has distinct urban and suburban regions with different population densities, built environments, and social dynamics. By conducting a comparative study within the same city, the research can effectively examine how neighborhood features and neighborly interactions influence community satisfaction in these distinct contexts. Furthermore, Beijing’s significance as the capital of China adds to the relevance and importance of the study. As a major cultural, economic, and political center, the findings from this research can potentially inform urban planning and policy decisions not only within Beijing but also in other cities facing similar challenges.

**Figure 2:**
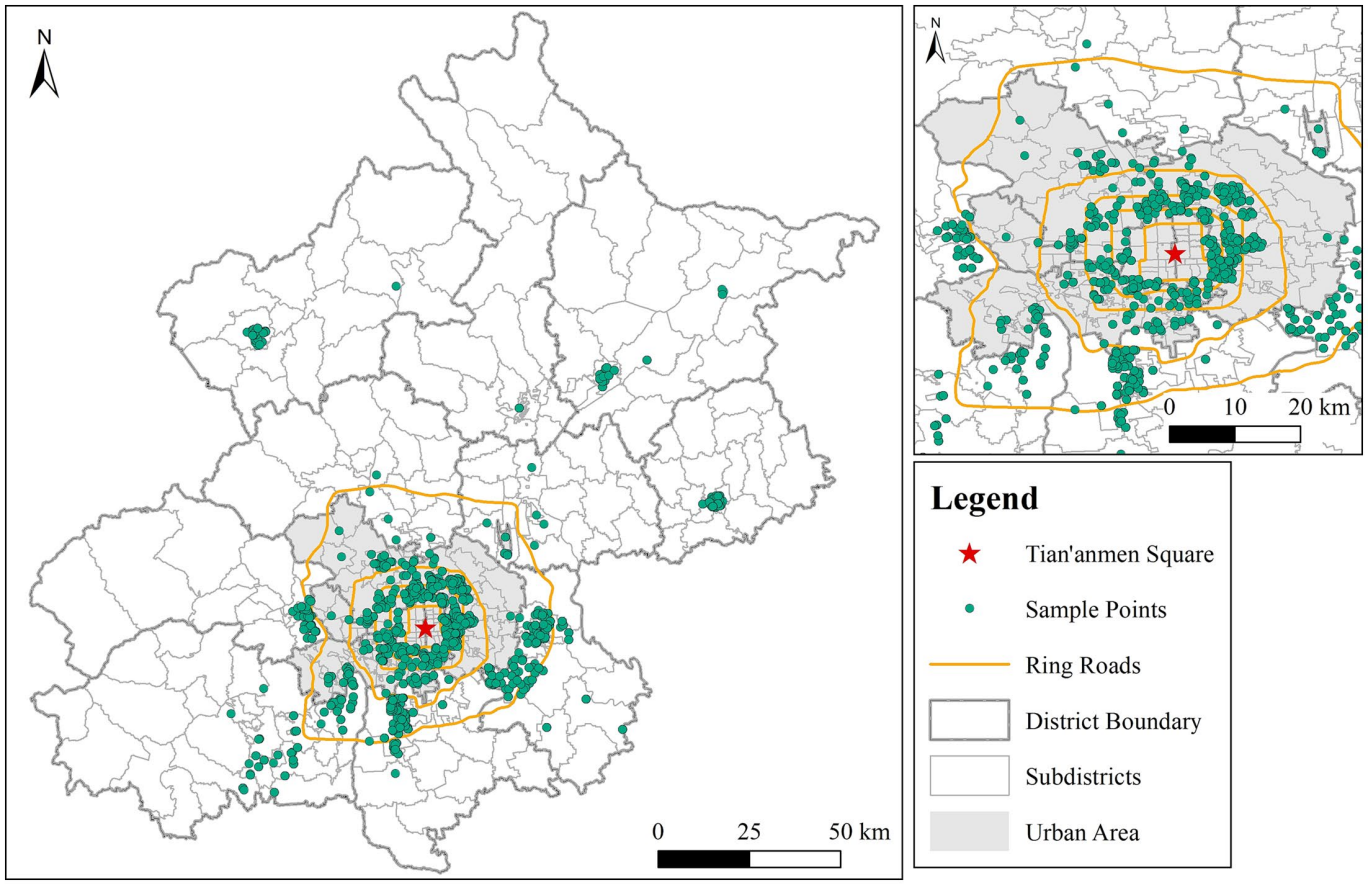
Spatial distribution of study area and surveyed respondents in Beijing.

Our analyses were drawn on a large-scale community satisfaction survey conducted in Beijing of City Health Examination Project implemented by the Ministry of Housing and Urban–Rural Development (MHURD) in China. The survey was carried out and completed in April 2018 and covered urban and suburban area (Figure 2). The questionnaire underwent a pilot study involving a sample of participants similar to our target population. This pilot study allowed us to assess the clarity and relevance of the questionnaire items and make necessary refinements to enhance its quality. To ensure data reliability and representativeness, we employed a multi-stage stratified proportional sampling method. The 16 districts of Beijing served as primary sampling units, with random selection of jie dao (primary sampling units, or towns) within each district. Within each jie dao, 5–15 housing estates (xiao qu) were chosen. Twenty-five surveyed respondents in each community were randomly selected with support from the local community council and invited to scan a QR code to access the questionnaire information collection system. A face-to-face questionnaire survey was then conducted by trained investigators. Because our survey covered many streets, it is representative of all types of residents’ community satisfaction in Beijing.

The residents’ community satisfaction survey in Beijing City Health Examination Project comprised of perceived quality of community surrounding environment (community satisfaction, environment quality, community service, community management, transportation convenience, and neighborhood interaction), respondents’ socioeconomic characteristics such as housing property, gender, age, educational, income, hukou, family members. Our survey also collected the geographic coordinates of respondents’ residences, with the support of a location-based service. The total number of questionnaires obtained in our survey was 4,011. After removing questionnaires with missing data, we finally have 4,009 effective surveys, with an effective rate of 95.45%.

### 3.2. Variables

Based on the literature review, a conceptual framework is developed to examine the heterogeneous influence of multiple neighborhood features on community satisfaction through neighborly interactions. Community satisfaction is the outcome variable measured by the survey question: “To what extent are you satisfied with the overall situation of your community?” Responses are quantified on a 5-point Likert scale ranging from 1 (very unsatisfied) to 5 (very satisfied).

Environmental quality in this study focuses on four key dimensions: greenery accessibility, air quality, noise, water waste. All respondents were asked, “How easy is it for you to access green spaces in nearby parks?” “To what extent do you think the air pollution in the city, such as PM2.5, is severe?” “To what extent do you think the noise pollution in the surrounding area is severe?” “Do you think the surrounding water body smells bad or changes color?” The response items comprised five options related to residents’ satisfaction: 1 (very inconvenient or similarly) to 5 (very convenient or similarly).

Community service in this study focuses on six key dimensions: daily shopping satisfaction, canteen satisfaction, shopping mall satisfaction, kindergarten satisfaction, health service satisfaction, and sports grounds satisfaction. Respondents were asked to rate their satisfaction levels with each of the six neighborhood dimensions on a 5-point Likert scale ranging from 1 (very unsatisfied or similarly) to 5 (very satisfied or similarly).

Community management in this study focuses on four key dimensions: garbage sorting, streetlamp maintenance, emergency respond to water/power outages, public fire potentials, well loss. Respondents were asked to rate their satisfaction levels with each of the variables, which were also developed on a five-point Likert scale ranging from 1 (very dissatisfied or similarly) to 5 (very satisfied or similarly).

Transportation convenience in this study focuses on six key dimensions: pedestrian condition, cycling condition, bus on-time, public transit transfer, metro setup, parking convenience. Variables depicting subjectively. These variables were developed by asking respondents’ satisfaction with them on a 5-point Likert scale ranging from 1 (very unsatisfied or similarly) to 5 (very satisfied or similarly).

Neighborhood interaction in this study focuses on three key dimensions: caring for vulnerable groups, minimum subsistence, and social activities. Respondents were asked to rate their satisfaction levels with each of the six neighborhood dimensions on a 5-point Likert scale ranging from 1 (very unsatisfied or similarly) to 5 (very satisfied or similarly).

Socioeconomic attributes were broadly included in the analysis not used as control variables, due to their high relevance to community satisfaction (52).

### 3.3. Methods

The study employs structural equations modeling (SEM) to investigate the heterogeneous influence of multiple neighborhood features on community satisfaction through neighborly interactions. SEM is a powerful multivariate statistical modeling technology that is used to test the theoretical model and hypothesis (69). Compared with other statistical techniques such as multiple regression, SEM can measure the latent constructs that cannot be observed directly, and can simultaneously analyze the relationship and intensity between multiple observed variables and latent variables. SEM consists of two parts: the structural model, which is used to describe the causal relationship between latent independent variables and latent dependent variables; and the measurement model, which is used to describe the linear relationship between latent variables and observed variables (69). There are two common forms of measurement equation, which can be expressed as follows:


(1) $$x=\Lambda_{x}\xi +\delta$$

(2) $$y=\Lambda_{y}\eta +\varepsilon$$

where x are the column vectors of the exogenous variables; $\Lambda_{x}$ are the factor loading matrices that associate the latent exogenous variables and observed variables; y are the column vectors of the endogenous variables; and $\Lambda_{y}$ are the factor loading matrices that correlate the latent endogenous variables and observed variables. ξ and η represent the latent exogenous and latent endogenous variables, respectively. δ and ε are error terms.

The structural equation can be written as follows:


(3) η=Bη+Γξ+ζ

where η and ξ are the column vectors of the endogenous and exogenous latent variables, respectively. B and Γ are factor loading matrices. ζ are error terms.

In this study, community satisfaction was used as the final endogenous variable in the model, and neighborhood interaction as the mediating variables. Environment quality, transportation convenience, community service, community management and socio-demographic variables are the exogenous variables. After confirmatory factor analysis (CFA) in the software AMOS 26.0, the latent variables including community service, community management, transportation convenience and neighborhood interaction were constructed with an acceptable fit and internal consistency (Table 1). The VIF values of explanatory variables are below, indicating a good fit between the model and the data.

**Table 1:** Description analysis of all variables.

Variables	Describe	Mean	SE
Community satisfaction	To what extent are you satisfied with the overall situation of your community? (1 = very dissatisfied, 5 = very satisfied)	4.11	0.872
Environment quality			
Greenery accessibility	How easy is it for you to access green spaces in nearby parks?(1 = Very inconvenient, 5 = Very convenient)	4.33	0.892
Air quality	To what extent do you think the air pollution in the city, such as PM2.5, is severe? (1 = very light, 5 = very severe)	3.99	1.071
Noise	To what extent do you think the noise pollution in the surrounding area is severe? (1 = very light, 5 = very severe)	3.82	1.089
Water waste	Do you think the surrounding water body smells bad or changes color? (1 = No smell or color change, 5 = Very serious)	4.2	0.956
Community service			
Daily shopping satisfaction	To what extent do you think it convenient to shop for daily necessities (convenience stores, grocery stores, delivery points) nearby? (1 = very inconvenient, 5 = very convenient)	4.42	0.849
Canteen satisfaction	To what extent are you satisfied with the community senior canteen/table? (1 = very unsatisfied, 5 = very satisfied)	3.32	1.749
Shopping mall satisfaction	To what extent are you satisfied with the large shopping centers and other facilities? (1 = not satisfied at all, 5 = very satisfied)	4.26	0.888
Kindergarten satisfaction	To what extent are you satisfied with the availability of affordable preschools? (1 = very dissatisfied, 5 = very satisfied)	3.97	1.105
Health service satisfaction	To what extent are you satisfied with the community health service center? (1 = very dissatisfied, 5 = very satisfied)	4.14	1.016
Sports grounds satisfaction	To what extent are you satisfied with the community sports facilities? (1 = very dissatisfied, 5 = very satisfied)	3.91	1.219
Community management			
Garbage sorting	To what extent are you satisfied with the level of garbage sorting in your community? (1 = very dissatisfied, 5 = very satisfied)	4.22	0.924
Streetlamp maintenance	To what extent are you satisfied with the management and maintenance of street lighting? (1 = very dissatisfied, 5 = very satisfied)	4.29	0.831
Emergency respond to water/power outages	To what extent are you satisfied with the emergency measures taken after water and power outage? (1 = Very dissatisfied, 5 = Very satisfied)	4.23	0.847
Public fire potentials	To what extent do you think there are fire safety hazards in the community? (1 = No hazards, 5 = Very serious)	4.02	0.936
Well loss	To what extent do you frequently observe missing or damaged manhole covers? (1 = Never, 5 = Very often)	4.4	0.879
Transportation convenience			
Pedestrian condition	To what extent are you satisfied with the walking environment? (1 = Very dissatisfied, 5 = Very satisfied)	4.21	0.903
Cycling condition	To what extent are you satisfied with the cycling environment? (1 = very dissatisfied, 5 = very satisfied)	4.13	0.957
Bus on-time	How punctual do you think the buses are in your city? (1 = very unpunctual, 5 = very punctual)	4.22	0.807
Public transit transfer	To what extent do you find it convenient to transfer between public transportation modes in your city? (1 = very inconvenient, 5 = very convenient)	4.36	0.786
Metro setup	To what extent are you satisfied with the community charging stations? (1 = very dissatisfied, 5 = very satisfied)	4.18	1.013
Parking convenience	To what extent do you think it convenient to park your car? (1 = very inconvenient, 5 = very convenient)	3.47	1.351
Neighborhood interaction			
Caring for vulnerable groups	To what extent do you think the city cares for vulnerable groups? (1 = not caring at all, 5 = very caring)	4.41	0.793
Minimum subsistence	To what extent are you satisfied with the level of minimum subsistence in your city? (1 = very dissatisfied, 5 = very satisfied)	4.15	0.938
Social activities	To what extent do your community organize various activities for residents to participate in? (1 = never, 5 = frequently)	4.27	0.819
Socioeconomic attributes		Frequency	Percentage
Housing property	0	902	22.5
	1 (1 = having property rights)	3,107	77.5
Gender	0	1,565	39
	1 (1 = female)	2,444	61
Age	< 20 years old	29	0.7
	20–29 years old	469	11.7
	30–39 years old	1,518	37.9
	40–49 years old	1,110	27.7
	50–59 years old	508	12.7
	60–69 years old	295	7.4
	> 70 years old	80	2
Education	Primary school and below	23	0.6
	Junior high school	199	5
	High school	454	11.3
	Junior college	1,266	31.6
	Undergraduate	1929	48.1
	Postgraduate and above	138	3.4
Income	Below 30,000 RMB	355	8.9
	30,000–49,000 RMB	342	8.5
	50,000–69,000 RMB	640	16
	70,000–99,000 RMB	831	20.7
	100,000–199,000 RMB	1,222	30.5
	200,000–299,000 RMB	394	9.8
	300,000–499,000 RMB	164	4.1
	Above 500,000 RMB	61	1.5
Hukou	0	227	5.7
	1 (1 = having local hukou)	3,782	94.3
		Mean	SE
Family members	What is the total number of members living together in your household?	3.32	1.21

## 4. Results

### 4.1. Descriptive statistics

The statistic analysis of the variables is showed in Table 1. The average of Community satisfaction reaches 4.11 with a standard deviation of 0.872. In terms of the environment quality, the average score of greenery accessibility is the highest, followed by water waste. Air quality, noise received relatively lower scores of 3.99 and 3.82, respectively. Air quality receives a relatively low score probably due to persistent issues with high levels of particulate matter (PM2.5) and other pollutants, stemming from factors like industrial emissions and meteorological conditions. The low noise rating may be a result of elevated noise levels in the city, primarily due to high population density, heavy traffic, and ongoing construction activities. In terms of the community service, the average score of daily shopping satisfaction is the highest, followed by shopping mall satisfaction and health service satisfaction. Kindergarten satisfaction, sports grounds satisfaction and canteen satisfaction received relatively lower scores of 3.97, 3.91, and 3.32, respectively. The high population density has led to a relatively insufficient supply of resources such as kindergartens, sports facilities, and cafeterias. Consequently, residents have rated these amenities lower in satisfaction. In terms of the community management, the average scores of all variables are relatively high, ranging from the range from the lowest in the public fire potentials to the highest in the well loss. In terms of the transportation convenience, the average score of all variable except or parking convenience are relatively high and significantly higher than 4. Parking convenience received the lowest scores of 3.47. Similarly, the high population density in Beijing has resulted in an insufficient supply of parking facilities. In terms of the neighborhood interaction, all indicators describing the neighborhood interaction have scores larger than 4.1. The average score of caring for vulnerable groups is the highest, followed by social activities. Minimum subsistence received the lowest scores of 4.15. This may be attributed to limited access to essential resources and services necessary for maintaining a basic standard of living, potential economic disparities, or challenges related to the affordability and availability of basic necessities.

Socio-economic characteristics considered in the survey included housing property, gender, age, education, income, hukou, and family numbers (Table 1). Among 4,009 respondents, 902 had not property rights and 3,107 had property rights. 1,565 were males and 2,444 were females, with a mean age value of 41.95. The most reported age of the respondents was in the 30–39 range. As for education, respondents with a degree above the undergraduate level were slightly over-represented (51.5% in total), followed by junior college. As for family’s monthly income, the most reported range was 100,000–199,000 RMB. Residents of Beijing with hukou accounted for 94.3% of the respondents, and 5.7% of the respondents were migrants. This meant that a majority of the respondents with local hukou had an owner-occupied house. The average number of family members living together in respondents’ household was 3.32 with a standard deviation of 0.872.

### 4.2. Association of multiple neighborhood features with community satisfaction

CFA was conducted to determine whether the measurement model was described correctly via data according to testing reliability and validity. Reliability measures the degree of data consistency or stability, and one of the most common evaluation methods is composite reliability (CR). All CR values presented in Table 2 are between 0.792 and 0.892, all above the standard of 0.70 indicating a high degree of internal consistency among the latent variables. To demonstrate convergent validity of the model, the average variance exacted (AVE) and factor loading were applied. As presented in Table 2, the standardized factor loadings of all items exceed the suggested threshold of 0.50 and are statistically significant for (*p* < 0.001). The AVE values of all constructs except for environment quality (AVE = 0.4888) were higher than the suggested benchmark score of 0.50. This indicates that each observed variable has high explanatory power. In conclusion, the result of the CFA showed that observed variables in measurement model could adapt to the data well.

The fitting indices also show an acceptable fitness. The $\chi^{2}/df\text{.}$ is 12.805 (values of 3 or less indicate a good fit), and other goodness-of-fit indices, such as RMSEA = 0.054 (values <0.05 indicate a good fit), SRMR = 0.0313 (values range from 0 to 1), CFI = 0.960 (values range from 0 to 1, and values >0.9 are acceptable), NFI = 0.956 and AGFI = 0.916 (values range from 0 to 1, and values >0.9 indicate a good fit).

The maximum likelihood estimation was used in evaluating the structure model. Indices such as $\chi^{2}/df\text{.}$ RMSEA, SRMR, CFI, NFI, and AGFI were selected to test the goodness of fit. What’s more, a bootstrapping procedure (with *N* = 5,000 bootstrap resamples) was applied to estimate indirect influence in the structural model and efficiently handle absence of multivariate normality. The results ( $\chi^{2}/df\text{.}$ = 9.569, RMSEA = 0.046, SRMR = 0.047, CFI = 0.951, NFI = 0.945, AGFI = 0.918) of overall model are presented in Table 3. Although the $\chi^{2}$ value is one of the most commonly used fitness indicators, it is often influenced by sample size and model complexity when the sample size is large. Considering that the more commonly used indicators SRMR and RMSEA perform well, and other fitting tables such as CFI, NFI, and AGFI also perform well, it can be considered that the overall model has a good fitting effect with the observed data. Regarding the grouping model, the fit indices also show an acceptable fitness.

**Table 2:** CFA results.

Latent variable	Manifest variable	Loading coefficient	*p*-value	Std. loading	CR	AVE
Environment quality	Noise	1		0.734	0.792	0.488
	Water waste	0.837	^***^	0.699		
	Air quality	0.84	^***^	0.626		
	Greenery accessibility	0.816	^***^	0.731		
Community service	Canteen satisfaction	1		0.697	0.886	0.565
	Community service satisfaction	0.646	^***^	0.775		
	Kindergarten satisfaction	0.706	^***^	0.779		
	Shopping mall satisfaction	0.554	^***^	0.760		
	Daily shopping satisfaction	0.463	^***^	0.667		
	Sports grounds satisfaction	0.822	^***^	0.822		
Community management	Public fire potentials	1		0.767	0.875	0.584
	Emergency response to water/power outages	0.989	^***^	0.839		
	Streetlamp maintenance	0.921	^***^	0.796		
	Garbage sorting	0.989	^***^	0.768		
	Well loss	0.779	^***^	0.637		
Transportation convenience	Parking convenience	1		0.712	0.892	0.582
	Metro setup	0.678	^***^	0.643		
	Public transit transfer	0.598	^***^	0.735		
	Bus on-time	0.646	^***^	0.769		
	Cycling condition	0.835	^***^	0.839		
	Pedestrian condition	0.805	^***^	0.857		
Neighborhood interaction	Minimum subsistence	1		0.855	0.813	0.593
	Caring for vulnerable groups	0.763	^***^	0.772		
	Social activities	0.687	^***^	0.673		

1. Model fitness *χ*^2^/df. 12.805, RMSEA 0.054, SRMR 0.0313, CFI 0.960, NFI 0.956, AGFI 0.916. 2. CR, composite reliability, AVE, average variance extracted. ^***^p-value < 0.001.

**Table 3:** SEM fitness.

	*χ*^2^/df.	RMSEA	SRMR	CFI	NFI	AGFI
Overall model	9.569	0.046	0.047	0.951	0.945	0.918
Grouping model	5.465	0.033	0.053	0.949	0.939	0.908

**Figure 3:**
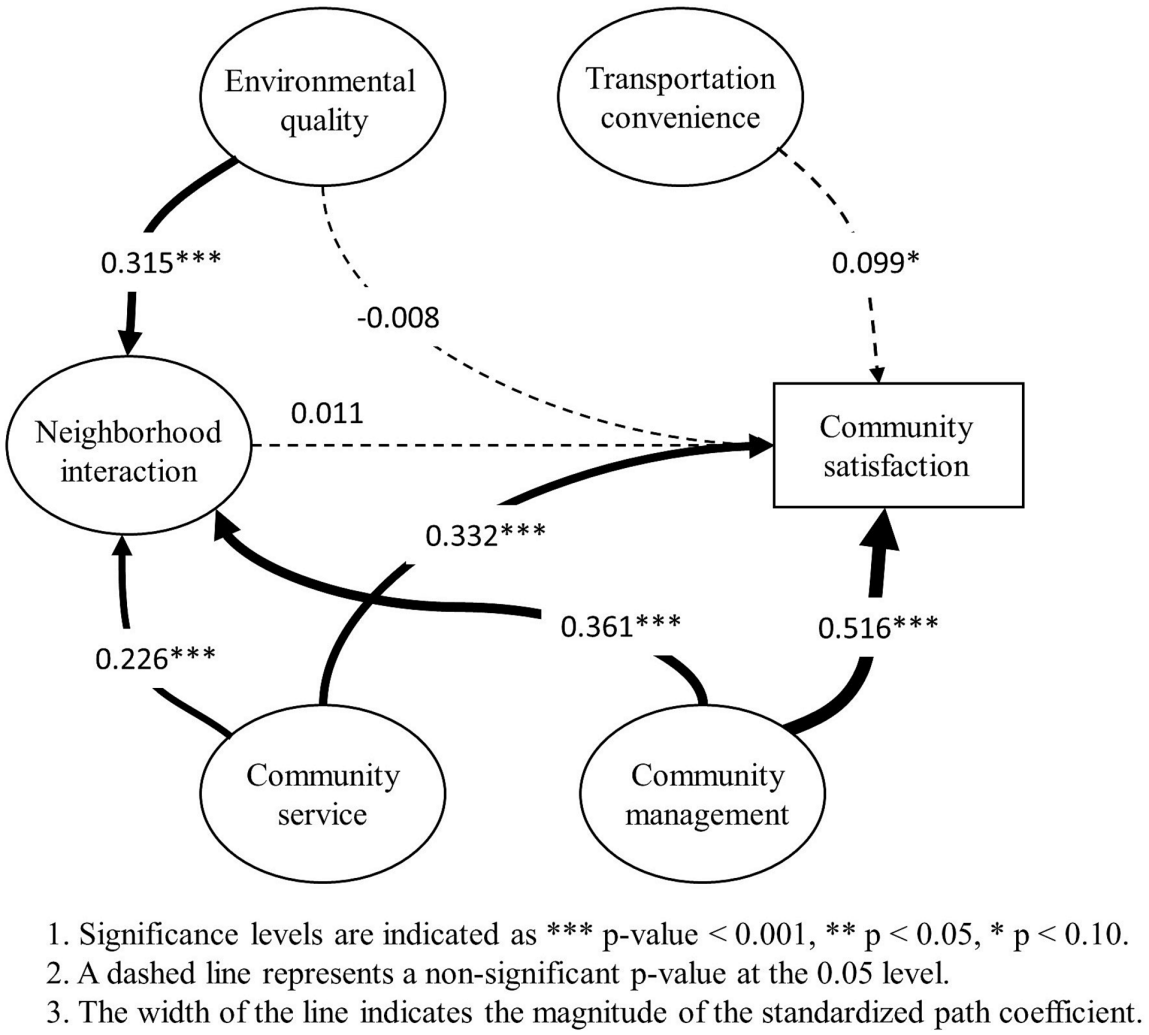
Association of multiple neighborhood features with community satisfaction.

The structural model that estimated the relationships among environment quality, community service, community management, neighborhood interaction, transportation convenience and socioeconomic attributes had an adequate fit (Table 4; Figure 3). In particular, environmental quality exerted a significant effect on neighborhood interaction (weight = 0.315, *p* < 0.001). However, the influence of that on Community satisfaction was not significant. Community service exerted a significant effect on both neighborhood interaction (weight = 0.226, *p* < 0.001) and community satisfaction (weight = 0.332, *p* < 0.001). Meanwhile, the strongest positively significant influence on both neighborhood interaction (weight = 0.361, *p* < 0.001) and community satisfaction (weight = 0.516, *p* < 0.001) was exerted by the determinant community management. Neighborhood interaction had no significant effect. Then, transportation convenience had relatively significant effect (weight = 0.099, *p* < 0.10). As for socioeconomic attributes, gender and family members exhibited influences on community satisfaction while housing property exerted a relatively remarkable negative effect.

**Table 4:** Structural model results: path coefficient between the constructs.

Path relation			Overall (*N* = 4,009)	Urban (*N*=)	Suburban
Environment quality	→	NI	0.315^***^	0.333^***^	0.304^***^
Environment quality	→	NS	−0.008	−0.043	0.014
Community service	→	NI	0.226^***^	0.224^***^	0.227^***^
Community service	→	NS	0.332^***^	0.294^***^	0.393^***^
Community management	→	NI	0.361^***^	0.317^***^	0.394^***^
Community management	→	NS	0.516^***^	0.353^***^	0.725^***^
NI	→	NS	0.011	0.054^**^	−0.036
Transportation convenience	→	NS	0.099^*^	0.286^***^	−0.141^*^
Housing property	→	NS	−0.015^**^	−0.017	−0.018^*^
Gender	→	NS	0.012^*^	0.015	0.01
Age	→	NS	0.006	−0.005	0.023^**^
Education	→	NS	0.010	0.011	0.016
Income	→	NS	−0.012	−0.011	−0.006
Family members	→	NS	0.016^**^	0.025^**^	0.006
Hukou	→	NS	0.000	0.011	−0.006

EQ, environment quality; CS, community service; CM, community management; NI, neighborhood interaction; NS, community satisfaction.

*** *p* < 0.001.

** *p* < 0.05.

* *p* < 0.10.

### 4.3. The heterogeneous effect of multiple neighborhood features on community satisfaction between urban and suburban communities

**Figure 4:**
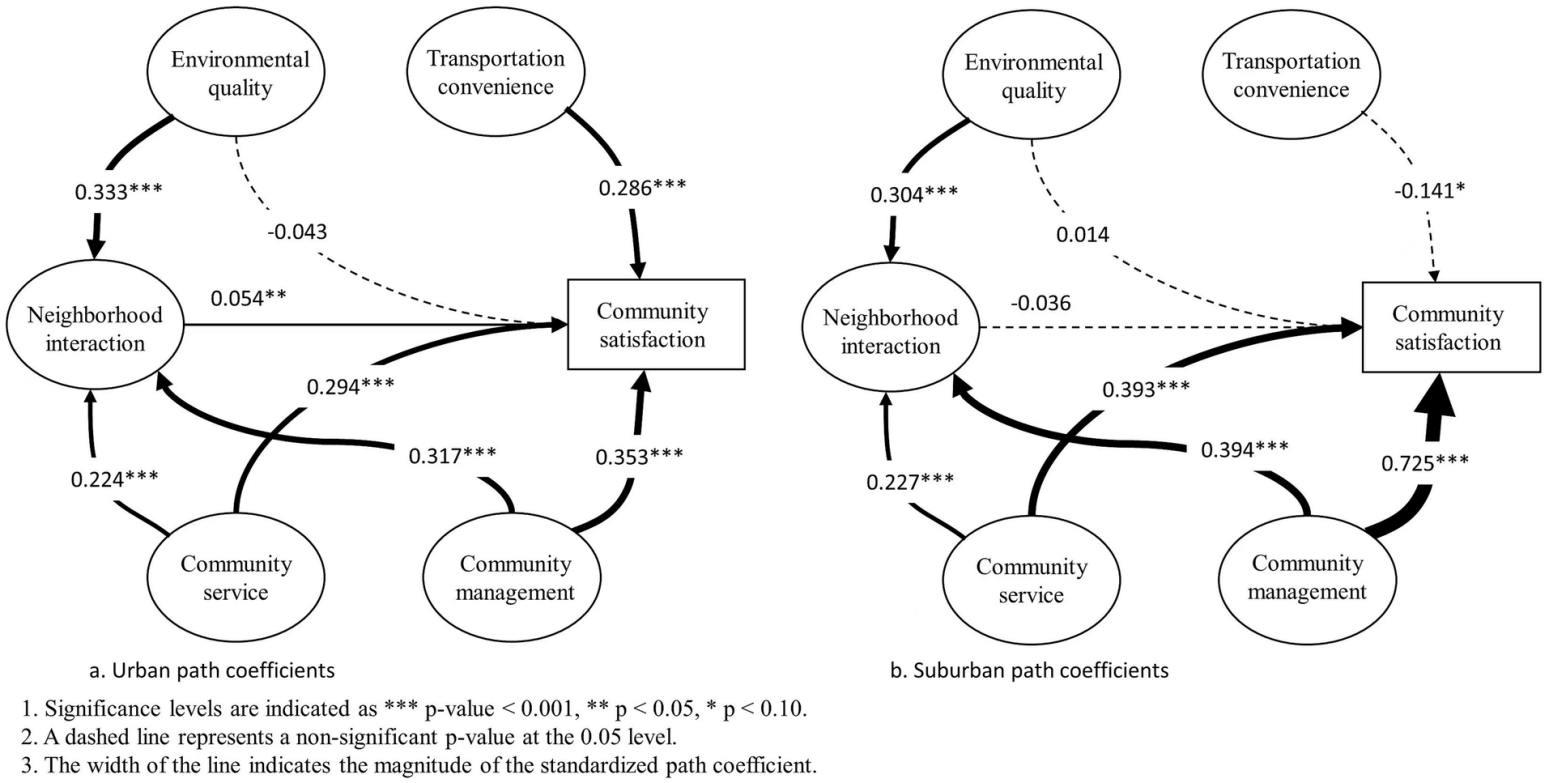
Association of multiple neighborhood features with community satisfaction in urban **(A)** and suburban **(B)** communities.

The influence of multiple neighborhood features on urban community satisfaction is slightly different from that of overall communities (Table 4; Figure 4A). In particular, environmental quality exerted a significant effect on neighborhood interaction (weight = 0.333, *p* < 0.001), stronger than that of overall communities. However, its influence on community satisfaction was insignificant. Community service exerted a significant effect on both neighborhood interaction (weight = 0.224, *p* < 0.001) and community satisfaction (weight = 0.294, p < 0.001). Meanwhile, the strongest positively significant influence on both neighborhood interaction (weight = 0.317, *p* < 0.001) and Community satisfaction (weight = 0.353, *p* < 0.001) was exerted by the determinant community management. Comparing with these effects of overall communities, these effects of urban communities were weaker. It is worth noting that neighborhood interaction had significant effect. Transportation convenience had more significant effect (weight = 0.286, *p* < 0.001) than that of overall communities (weight = 0.099, *p* < 0.10). As for socioeconomic attributes, only family members (weigh = 0.025, *p* < 0.05) exhibited a relatively remarkable positive influence on Community satisfaction.

The influence of multiple neighborhood features on suburban community satisfaction is similar to that of overall communities (Table 4; Figure 3). In detail, environment quality exerted a significant effect on neighborhood interaction (weight = 0.304, *p* < 0.001), weaker than that of overall communities. However, the influence of that on Community satisfaction was not significant. Community service exerted a significant effect on both neighborhood interaction (weight = 0.227, *p* < 0.001) and Community satisfaction (weight = 0.393, *p* < 0.001). Meanwhile, the strongest positively significant influence on both neighborhood interaction (weight = 0.394, *p* < 0.001) and Community satisfaction (weight = 0.725, *p* < 0.001) was exerted by the determinant community management. Comparing with these effects of overall communities, these effects of urban communities were stronger. However, transportation convenience exhibited a remarkable negative influence on Community satisfaction. As for socioeconomic attributes, only age exhibited a relatively remarkable positive influence on Community satisfaction (weigh = 0.023, *p* < 0.05).

The influence of multiple neighborhood features on suburban community satisfaction is different between urban and suburban (Table 4; Figure 4). It shows that community service and community management have a significant direct and positive influence on Community satisfaction in both urban and suburban communities while environment quality have an indirect and positive influence. These three latent variables are partially mediated by neighborhood interaction. In addition, neighborhood interaction and community satisfaction are more influenced by community service and community management in suburban communities whereas neighborhood interaction is more subject to the influence of environment quality in urban communities. Environment quality is not sensitive to the direct impact on community satisfaction in both urban and suburban communities. In the urban community, the direct influence of neighborhood interaction and transportation convenience on community satisfaction is significant and positive. In the suburban communities, the direct influence of neighborhood interaction is not sensitive to community satisfaction and transportation convenience on community satisfaction is significant and negative.

## 5. Discussion

The findings of this study contribute valuable insights into the understanding of how neighborhood features influence community satisfaction, with a focus on the heterogeneity observed between urban and suburban communities in Beijing. The use of structural equation models and a large-scale survey involving 4,009 respondents allowed for a comprehensive analysis of the data and provided robust evidence for the heterogeneous role of neighborhood features in shaping community satisfaction. This study contributes the empirical evidence to urban planning, community development, or policy formulation by comparing community satisfaction between urban and suburban.

One of the key findings of this study is the significant influence of community service and community management on community satisfaction, indicating the importance of effective service delivery and well-organized management structures in fostering positive perceptions among residents. Numerous studies in the field of urban planning and community development have emphasized the critical role of effective service delivery in enhancing community satisfaction (12, 70, 71). Services such as healthcare, education, public safety, and recreational facilities have been shown to contribute significantly to residents’ well-being and contentment with their community. A well-organized and responsive service system can address residents’ needs and improve their overall quality of life, leading to higher levels of community satisfaction. According to Cao and Wang, the availability of quality community services, such as healthcare facilities, educational institutions, recreational centers, and public spaces, plays a crucial role in enhancing residents’ overall satisfaction with their living environment (46). Likewise, efficient community management, encompassing maintenance, security, and dispute resolution, ensures a well-functioning and harmonious community, further contributing to resident contentment.

Then, the study revealed the differential effects of specific neighborhood features in urban and suburban communities and essentially it suggests how particular aspects or characteristics of neighborhoods impact residents in different contexts. In urban areas, transportation convenience emerges as a key factor positively impacting community satisfaction. According to Buys and Miller, the ease of accessibility to public transportation and efficient mobility options play a crucial role in enhancing residents’ overall satisfaction with their living environment (72). An efficient transportation network not only reduces commuting time and enhances connectivity but also improves access to essential amenities and services, enhancing the overall quality of life in urban communities (17). Besides, sustainable and convenient transportation options, such as public transit, cycling lanes, and pedestrian-friendly infrastructure, have the potential to create cleaner and less congested urban areas. These improvements not only reduce pollution and congestion but also contribute to the development of healthier and more pleasant cities. On the contrary, in suburban communities, the presence of housing property has a negative effect on community satisfaction. This result suggests that issues related to housing property, such as maintenance, affordability, or structural conditions, may be contributing to lower levels of satisfaction among suburban residents. Policymakers and community planners need to focus on addressing these housing-related concerns to improve resident satisfaction in suburban areas.

Moreover, the study highlights the pivotal role of neighborly interaction in mediating the relationship between multiple neighborhood features and community satisfaction. It means neighborhood features alone may not directly determine community satisfaction. Instead, these features have their effects channeled through the quality of neighborly interactions. In other words, neighborly interactions help explain why certain neighborhood features lead to higher or lower community satisfaction. The finding accords to conclusions made by previous studies that social cohesion and a sense of community are closely linked to residents’ satisfaction with their neighborhoods. Neighborly interactions, such as socializing with neighbors, participating in community events, and collaborating on local projects, contribute to the development of social ties and cohesion. Strong social ties and positive relationships among neighbors create a sense of belonging and support within the community, enhancing residents’ contentment with their living environment (64). The significance of neighborly interactions in shaping community satisfaction underscores the importance of promoting social cohesion and community engagement initiatives (30). Encouraging community events, neighborhood gatherings, and collaborative projects can foster a strong sense of community identity and belonging, ultimately leading to higher levels of satisfaction among residents.

While this study provides valuable insights, there are some limitations to acknowledge. The cross-sectional design restricts causal inferences due to the lack of long-term follow-up investigation, and future longitudinal studies could offer a deeper understanding of the dynamics between neighborhood features and community satisfaction over time. Additionally, the study’s focus on Beijing may limit the generalizability of the findings to other urban and suburban settings. Future research should expand the coverage and representation of the sampled cities to conduct similar investigations, achieving a broader perspective on community satisfaction factors and their variations across diverse urban and suburban contexts. Thirdly, due to the difference in questionnaire quantity between urban and suburban, the evaluation of the perceived community satisfaction variables could be overestimated or underestimated. Future research should focus on urban or suburban to accurately understand their community satisfaction and its influencing factors.

## 6. Conclusion

Community satisfaction plays a pivotal role in shaping urban planning, community development, and policy formulation. However, our understanding of how diverse neighborhood features impact satisfaction remains incomplete. This study delves into the influence of neighborhood features on community satisfaction, with a particular focus on the variations between urban and suburban communities. Our approach involves the application of structural equation models to analyze data from a comprehensive survey conducted in Beijing, encompassing 4,009 respondents.

The findings underscore that community services and effective community management have substantial positive effects on community satisfaction, primarily mediated through neighborly interactions. Additionally, in urban areas, transportation convenience emerges as a key driver of community satisfaction, whereas in suburban communities, no housing property negatively affects satisfaction. These outcomes highlight the nuanced impact of neighborhood features on community satisfaction, providing valuable insights for tailoring urban planning and policies to meet the unique characteristics and needs of different communities. These findings have important implications for urban planning and policy interventions. Recognizing the heterogeneity in neighborhood influences on community satisfaction, it is crucial to develop customized approaches that address the specific characteristics and needs of different community types. Urban planners and policymakers should prioritize the provision of high-quality community services and effective community management practices to enhance community satisfaction. In urban areas, investments in transportation infrastructure and policies that promote accessibility can play a key role in fostering community satisfaction. In suburban communities, efforts should be made to address housing-related issues and improve housing conditions to mitigate the negative impact on community satisfaction. These endeavors would contribute significantly to the achievement of the Sustainable Development Goals (SDGs) and other pertinent international initiatives.

In conclusion, this comparative study sheds light on the heterogeneous influence of multiple neighborhood features on community satisfaction, with neighborly interactions playing a significant mediating role. The findings underscore the importance of considering the specific characteristics and needs of different community types in urban planning and policy interventions. By addressing the identified factors, such as community service, community management, transportation convenience, and housing property, policymakers and urban planners can contribute to fostering community satisfaction, enhancing well-being, and promoting social cohesion within society.

## Data availability statement

The raw data supporting the conclusions of this article will be made available by the authors, without undue reservation.

## Author contributions

HJ: Conceptualization, Data curation, Funding acquisition, Methodology, Project administration, Writing – original draft, Writing – review & editing. RC: Data curation, Formal Analysis, Investigation, Writing – original draft. DY: Conceptualization, Formal Analysis, Methodology, Validation, Writing – original draft. ZH: Conceptualization, Formal Analysis, Methodology, Visualization, Writing – original draft. JS: Funding acquisition, Resources, Validation, Writing – review & editing.
